# Rapidly Dissolving Microneedles for the Delivery of Steroid-Loaded Nanoparticles Intended for the Treatment of Inflammatory Skin Diseases

**DOI:** 10.3390/pharmaceutics15020526

**Published:** 2023-02-04

**Authors:** Hala Dawud, Aiman Abu Ammar

**Affiliations:** Department of Pharmaceutical Engineering, Azrieli College of Engineering Jerusalem, 26 Yaakov Shreibom Street, Ramat Beit HaKerem, Jerusalem 9103501, Israel

**Keywords:** steroids, inflammatory skin diseases, controlled release, dissolving microneedles, PLGA (poly lactic-co-glycolic acid), nanoparticles

## Abstract

Drug delivery through the skin has immense advantages compared to other routes of administration and offers an optimal way to treat inflammatory skin diseases, where corticosteroids are the cornerstone of topical therapy. Still, their therapeutic efficiency is limited due to inadequate skin permeability, potential side effects, and reduced patient compliance. To overcome these drawbacks, we propose a drug delivery system consisting of dexamethasone (DEX)-loaded poly(lactic-co-glycolic acid) (PLGA) nanoparticles (NPs) incorporated in sodium alginate (SA) microneedles (MNs) as a minimally invasive dosage form for controlled drug release. Drug-loaded PLGA NPs were prepared by a nanoprecipitation method with a high encapsulation yield. They exhibited a controlled release pattern over 120 h. A modified vacuum-deposition micromolding method was used to load the obtained DEX-NPs into the tips of dissolving MNs. The NP-MNs showed improved insertion capabilities into the skin-simulant parafilm model and enhanced mechanical strength when tested against different static forces compared to their counterparts (SA-MNs). The results of an MN dissolution study following application to ex vivo chicken skin and agarose gel indicate that the NP-loaded segments of MNs dissolve within 15 s, in which the NPs are released into the skin. Taken together, the incorporation of DEX-NPs into SA-MNs could be a promising approach to bypass the limitations of conventional topical treatment of skin diseases, allowing for self-administration, increased patient compliance, and controlled drug release.

## 1. Introduction

The use of microneedles (MNs) is an innovative and minimally invasive approach that has been developed in the last two decades to deliver active pharmaceutical substances through the skin by overcoming the main skin barrier, the stratum corneum (SC). MNs are micron-sized structures in the height range of 50 to 1500 μm allowing for painless penetration into the skin via the SC, viable epidermis, and upper dermis without contacting nerve fibers or blood vessels. Drug-loaded MNs create micron-sized pores in the skin to enhance drug delivery to the different layers of the skin for local or systemic treatment. Thus, numerous factors should be taken into account when designing MN delivery, such as geometry, needle height, thickness and tip radius, base diameter, needle density, and MN material. Compared to traditional transdermal delivery systems, MNs possess several advantages, including enhanced bioavailability and drug permeation, self-administration, and improved patient compliance [1,2].

Different types of MNs were developed, such as solid, hollow, coated, dissolving, and hydrogel-forming MNs, which differ in their delivery strategy, fabrication methods, geometry, and the materials used in their manufacturing. Several constituent materials, such as metals, ceramics, sugars, silicon, and biodegradable polymers, are widely used to fabricate MNs due to their advantages, including low cost, simple manufacturing, biocompatibility, stability, folding ability, and favorable drug loading capacity [3,4,5].

Dissolving MNs can encapsulate therapeutic agents and release their content once inserted into the skin. Sodium alginate (SA) is widely used in the pharmaceutical and biomedical fields for the fabrication of wound dressings, drug encapsulation, and coating due to its unique properties, such as biocompatibility, biodegradability, and low cost [6]. The chemical structure of SA includes free hydroxyl and carboxyl groups distributed along the chain of the polymer, making it highly soluble in water [7]. Since the mechanism of dissolving MNs is to release the drug once the tips are dissolved due to contact with the interstitial fluids, SA offers good applicability in dissolving MNs fabrication.

PLGA is one of the most frequently used polymers for drug delivery due to its FDA approval, biodegradability, and biocompatibility [8,9]. Nanoparticles (NPs) are widely used for drug delivery owing to their size, which allows an improved biodistribution of the entrapped drug, as well as the capability to release the drug to its specific site in a controlled manner. PLGA NPs exhibit a wide range of degradation times depending on PLGA properties, such as molecular weight and the ratio between its constituent monomers, making it one of the best candidates for controlled drug delivery systems [10,11].

Since MNs can penetrate the skin easily and provide a site-specific delivery in a minimally invasive manner, they can serve as a vehicle for the treatment of several skin diseases, such as psoriasis, dermatitis, eczema, acne, and skin cancer [12]. Topical corticosteroids are one of the most widely used treatment modalities for inflammatory skin diseases. They are considered the first-line treatment because of their therapeutic potential, being vasoconstrictors, anti-inflammatory, immunosuppressive, and anti-proliferative drugs [13,14]. Topical corticosteroids can be formulated in different dosage forms, such as creams, ointment, and lotions. However, these dosage forms could lead to poor patient compliance due to their greasy texture, odor, stickiness, dosage frequency, and potential systemic and local side effects [15,16].

Therefore, a nano-delivery system may be advantageous by facilitating direct drug delivery at the site of interest and the release of the drug in a sustained manner. Hence, the purpose of this study is to achieve controlled drug release by the incorporation of a model drug, dexamethasone, into PLGA nanoparticles and embedding it in rapidly dissolving microneedles made of sodium alginate (NP-MNs) for improved treatment of skin diseases, in terms of both therapeutic effect and patient compliance. Blank sodium alginate microneedles (SA-MNs) were prepared and used as controls.

## 2. Materials and Methods

### 2.1. Materials

Dexamethasone (Alfa Aesar) and sodium alginate (Fisher Chemical) were purchased from Holland-Moran Inc. Israel. PLGA-Purasorb PDLG 5010 (50:50) was donated by Corbion Purac (Gorinchem, The Netherlands). Solutol HS 15 was supplied by BASF (Ludwigshafen, Germany). Silicone MPatch microneedle templates were purchased from Micropoint Technologies Pte Ltd. (Pioneer Junction, Singapore) and were pyramidal in shape with a dimension of 10 × 10 needle array, 200 µm base, 500 µm height, and 500 µm pitch. Phosphate buffer saline (PBS) was purchased from Hyclone Laboratories. Organic solvents were obtained from Sigma-Aldrich (Rehovot, Israel).

### 2.2. Preparation of DEX-Loaded PLGA NPs

DEX-loaded NPs were prepared using the nanoprecipitation method with modification [17,18]. Briefly, 2 mg of DEX and 6 mg of PLGA were dissolved in 1 mL acetone. The organic phase was added rapidly into an aqueous phase (2 mL) containing 0.5% (*w*/*v*) solutol HS 15, which was continuously stirred at room temperature (25 °C, 900 rpm) for 24 h to evaporate the organic solvent, followed by adjustment of the formulation volume to 2 mL by distilled water. The vials were wrapped with aluminum foil to avoid drug degradation due to light exposure. Blank PLGA NPs were prepared using the same method but without adding DEX at any stage of the preparation.

### 2.3. Physicochemical Characterization of DEX NPs

Particle size (hydrodynamic diameter), polydispersity index (PDI), and zeta potential were determined by the dynamic light scattering (DLS) method using a Malvern ZetaSizer nano-ZS laser particle size distribution analyzer (Zetasizer Pro, Malvern Instruments, Malvern, UK). Samples were diluted at 1:100 (*v*/*v*) with water and added to folded capillary zeta cells (DTS1070) to analyze at a 90° angle at room temperature (25 °C). For each sample, the mean value ± s.d. of three determinations was established.

### 2.4. Determination of Drug Encapsulation Efficiency and Loading Content

Encapsulation efficiency (EE) and drug loading content (DLC) were determined by first separation of the unloaded drug (free DEX) by filtration/centrifugation using an Amicon^®^ Ultra-15 (molecular weight cut-off 100 kDa) centrifugal filter unit (Merck Millipore Ltd., Burlington, MA, USA). The formulation samples were added to the upper chamber of the Amicon^®^ tube and then washed with an equal volume of distilled water 3 times by centrifugation at 3000 rpm for 1 min each. Finally, the amount of free DEX was determined from the filtrate using a UV-Vis spectrophotometer (GeneQuant 1300; Biochrom, Cambridge, UK) at a wavelength of 242 nm [19]. The concentrations of the calibration curve ranged from 0 to 25 μg/mL (Figure S1). The EE and DLC were calculated using the following equations:

(1) $$\mathrm{EE}\;\left(\%\right)=\frac{\mathrm{amount}\;\mathrm{of}\;\mathrm{DEX}\;\mathrm{fed}\;\mathrm{initially}\;-\;\mathrm{amount}\;\mathrm{of}\;\mathrm{free}\;\mathrm{DEX}\;}{\mathrm{amount}\;\mathrm{of}\;\mathrm{DEX}\;\mathrm{fed}\;\mathrm{initially}}\times 100$$

(2) $$\mathrm{DLC}\;\left(\%\right)=\frac{\mathrm{amount}\;\mathrm{of}\;\mathrm{DEX}\;\mathrm{fed}\;\mathrm{initially}\;-\;\mathrm{amount}\;\mathrm{of}\;\mathrm{free}\;\mathrm{DEX}\;}{\mathrm{amount}\;\mathrm{of}\;\mathrm{formulation}\;\mathrm{components}}\times 100$$

Afterward, the nanoparticles from the upper chamber of the Amicon^®^ tube were centrifuged at 3000 rpm for 1 min to remove the polymeric debris, and polymeric debris was analyzed to ensure that no drug residues were present.

### 2.5. Attenuated Total Reflectance–Fourier Transform Infrared Spectroscopy (ATR-FTIR) Measurements

ATR-FTIR spectra were measured by a PerkinElmer Spectrum 100S spectrometer equipped with a universal ATR sampling accessory. For each spectrum, 4 scans were collected with a resolution of 2 cm^−1^, and the scan range was between 4000 and 650 cm^−1^.

### 2.6. Differential Scanning Calorimetry (DSC) and X-ray Diffraction (XRD)

The thermal behavior of DEX-loaded nanoparticles, PLGA, dexamethasone, and the physical mixture was analyzed by a DSC 1 Star System equipped with Star Software (Mettler Toledo, Greifensee, Switzerland) and a DSC131 Evo (SETARAM Instrumentation, Caluire-etCuire, Lyon, France). Weighed samples of 6–11 mg were placed into 100 μL aluminum crucibles and the samples were scanned from 25 °C to 300 °C at a constant heating rate of 20 °C/min.

X-ray powder diffraction measurements were performed on the D8 Advance diffractometer with a LYNXEYE-XE-T detector (Bruker AXS, Karlsruhe, Germany) operating in 1D mode. Low-background quartz sample holders were carefully filled with the powder samples. XRD patterns within the range 2° to 75° 2θ were recorded at room temperature using CuKα radiation (λ = 1.5418 Å) with the following measurement conditions: tube voltage of 40 kV, tube current of 40 mA, step-scan mode with a step size of 0.02° 2θ, and counting time of 0.5 s/step.

### 2.7. Fabrication of SA-MNs and NP-MNs

**Figure 1:**
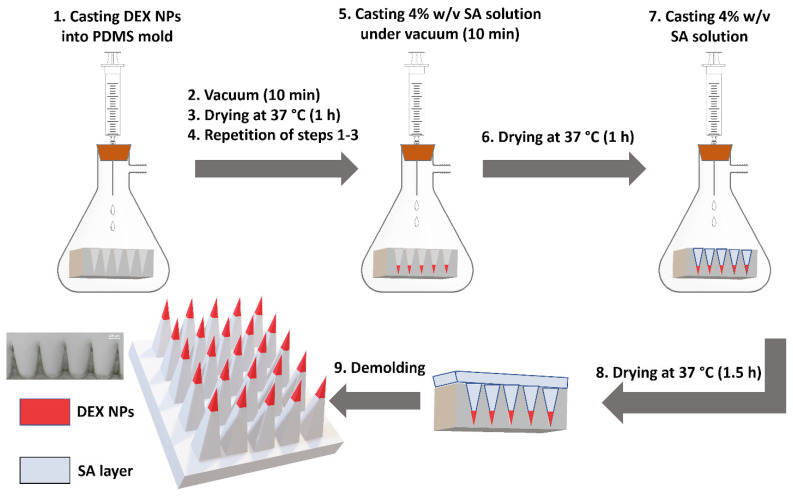
Preparation scheme of NP-loaded MNs by a vacuum-deposition micromolding method using a polydimethylsiloxane (PDMS) microneedle mold.

**Scheme 1:**
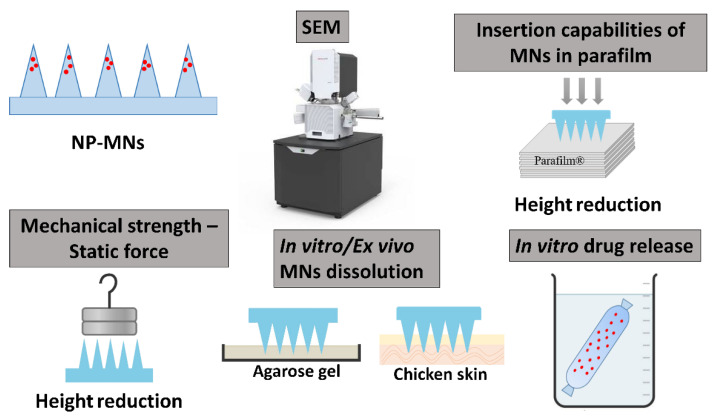
Schematic representation of characterization of NP-MNs.

NP-MNs were prepared using a vacuum-deposition micromolding method [20] with a modification, as shown in Figure 1. This method was chosen and adopted after applying the optimization process that included the use of different approaches, such as centrifugation. In brief, A PDMS micromold was placed into a vacuum flask, and 150 µL of DEX-loaded NPs were injected into the micromold surface, followed by a vacuum for 10 min and drying at 37 °C for 1 h. This process was repeated one more time, and then 150 µL of 4% *w*/*v* SA solution was injected under vacuum through the septum into the micromold surface, held for 10 min, and allowed to dry at 37 °C for 1 h. Afterward, 100 µL of 4% *w*/*v* SA solution was added to form the base of MNs and dried at 37 °C for 1.5 h before being demolded. SA-MNs were fabricated by the same method as NP-MNs without adding NPs at any stage of the preparation. The MNs were characterized as depicted in Scheme 1.

### 2.8. Morphological Characterization of MNs

Morphological evaluation of NP-MNs and SA-MNs was performed by a scanning electron microscope (SEM, Apreo 2; Thermo Fisher Scientific, Waltham, MA, USA).

### 2.9. Insertion Capabilities of MN Array in Parafilm

SA-MNs and NP-MNs were manually pushed into 8 stacked parafilm layers as a skin stimulant for 30 s and observed under a stereomicroscope (Olympus-SZ61, Tokyo, Japan) to study the insertion efficiency and MN insertion depth [21]. The insertion was expressed in the number of pores created in each parafilm layer, and the rate of change of the height (µm) of 10 randomly selected MNs was calculated and plotted as the percentage of reduction in MN height [22].

### 2.10. Evaluation of Mechanical Properties

The mechanical strength of MNs against static forces was measured by placing different weights against the MNs. Briefly, SA-MNs and NP-MNs were placed upward, onto which weights of 50 g, 500 g, and 1000 g were placed gently on the top of each patch, respectively. After 5 min, the weights were removed, and morphological changes were evaluated by a HAYEAR 4K UHD microscope camera. The rate of change of the height (µm) of the MNs was calculated and plotted as the percentage of reduction in MN height [23,24].

### 2.11. Dissolution Test of MNs In Ex Vivo Chicken Skin and Agarose Gel

The MN arrays were applied on ex vivo chicken skin obtained from a local slaughterhouse, and agarose gel 3% (*w*/*v*) served as a skin simulant. A weight of 500 g was placed above the arrays, and MNs were removed after predetermined times of 15, 45, and 120 s. The MNs were imaged before and after dissolution using a microscope camera [25].

### 2.12. In-Vitro Drug Release Studies

DEX NPs samples (200 µL) were placed into dialysis bags with a molecular cut-off of 8000 Da, soaked in 10 mL of PBS pH 7.4, and maintained at 37 °C (50 rpm) in a rotary incubator. At predetermined time intervals, 0.5 mL of the release medium was sampled and was replenished immediately with the same volume of fresh prewarmed PBS (37 °C), maintaining sink condition throughout the experiment. Then, DEX concentration was determined using a UV–Vis spectrophotometer at a wavelength of 242 nm [26]. The concentrations of DEX in PBS (pH = 7.4) ranged from 0 to 20 µg/mL (Figure S2). The experiment was performed in four replicates.

## 3. Results and Discussion

### 3.1. Preparation and Characterization of DEX-Loaded NPs

In this study, DEX-loaded PLGA nanoparticles were prepared by nanoprecipitation with a mean particle size of 93.7 nm, narrow size distribution (PDI < 0.3), and negative zeta potential values. DEX was successfully incorporated into the PLGA NPs, exhibiting an encapsulation efficiency of 80% and adequate drug loading content of 9.1% (Table 1). Previous studies have shown that the drug loading content of most of the existing nanomedicines is less than 10% [27]. Taking into consideration that a limited amount of drug can be loaded into the microneedles [2], it is crucial to obtain satisfactory drug loading into NPs to enhance the therapeutic efficacy.

#### 3.1.1. ATR-FTIR Measurements

**Figure 2:**
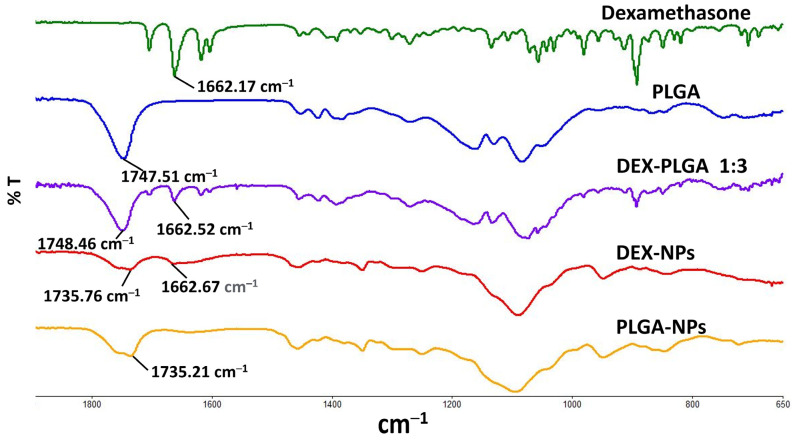
FTIR spectra of DEX, PLGA, physical mixture, DEX-NPs, and blank PLGA-NPs.

ATR-FTIR spectra of DEX, PLGA, physical mixture, DEX-NPs, and blank-NPs were measured (Figure 2). In the spectrum of DEX, the characteristic peaks were observed at 1704.01, 1662.17, and 1617.57 cm^−1^, in agreement with previous reports [28,29]. These peaks were attributed to the stretching vibrations of C=O and C=C (in a ring) double bond framework conjugated to C=O bonds in DEX. The characteristic peak of PLGA was identified at 1747.51 cm^−1^ due to the ester group [22]. The spectrum of the physical mixture exhibited relatively similar peaks of DEX and PLGA, including the band at 893 cm^−1^ that corresponds to the axial deformation of the C-F group in DEX. The spectrum of the DEX-NPs evidenced the contribution of the organic groups of PLGA and solutol HS 15 and did not fully demonstrate the bands equivalent to DEX. This was attributed to the superposition of the bands from all the NP components and could be further explained by the loading content of DEX as well as the relatively low peak intensity. The spectra of blank-NPs and DEX-NPs showed a peak at 1735 cm^−1^, which was ascribed to the used surfactant, solutol HS 15 (Figure S3), and overlapped with the C=O ester stretching vibration of PLGA. The peak corresponding to C=O stretch in DEX was observed at 1662.67 cm^1^ in the case of drug-containing NPs, where it was absent in the spectrum of blank NPs, suggesting the successful entrapment of DEX within PLGA NPs with no significant shift that would indicate chemical interaction between the drug and the polymer.

#### 3.1.2. DSC and XRD

**Figure 3:**
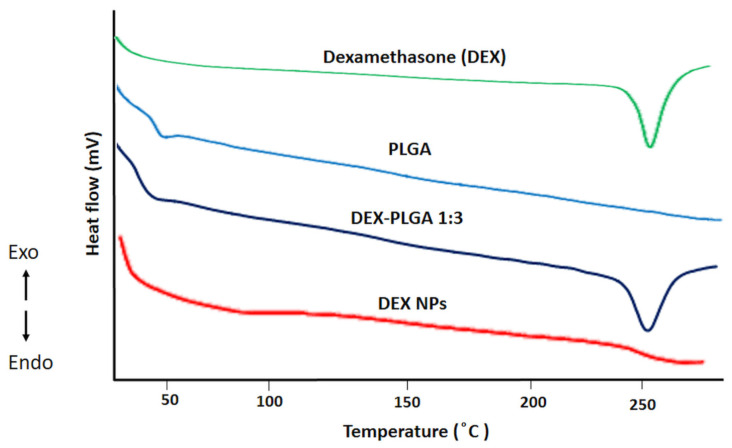
DSC thermograms of Dexamethasone, PLGA, their physical mixture (1:3 ratio), and DEX-NPs.

Thermal analysis using DSC was performed for DEX, PLGA, physical mixture, and DEX-NPs (Figure 3). The DSC thermogram of PLGA exhibited an endothermic peak at 50 °C depicting its glass transition temperature [30]. The thermal behavior of DEX was characterized by an endothermic peak at 255 °C, which is attributed to the melting point of the crystalline drug [31,32]. As shown in Figure 3, no endothermic melting peak was determined for DEX NPs, which may be because DEX was dispersed at a molecular state within the nanoparticles in an amorphous or disordered crystalline state [33].

**Figure 4:**
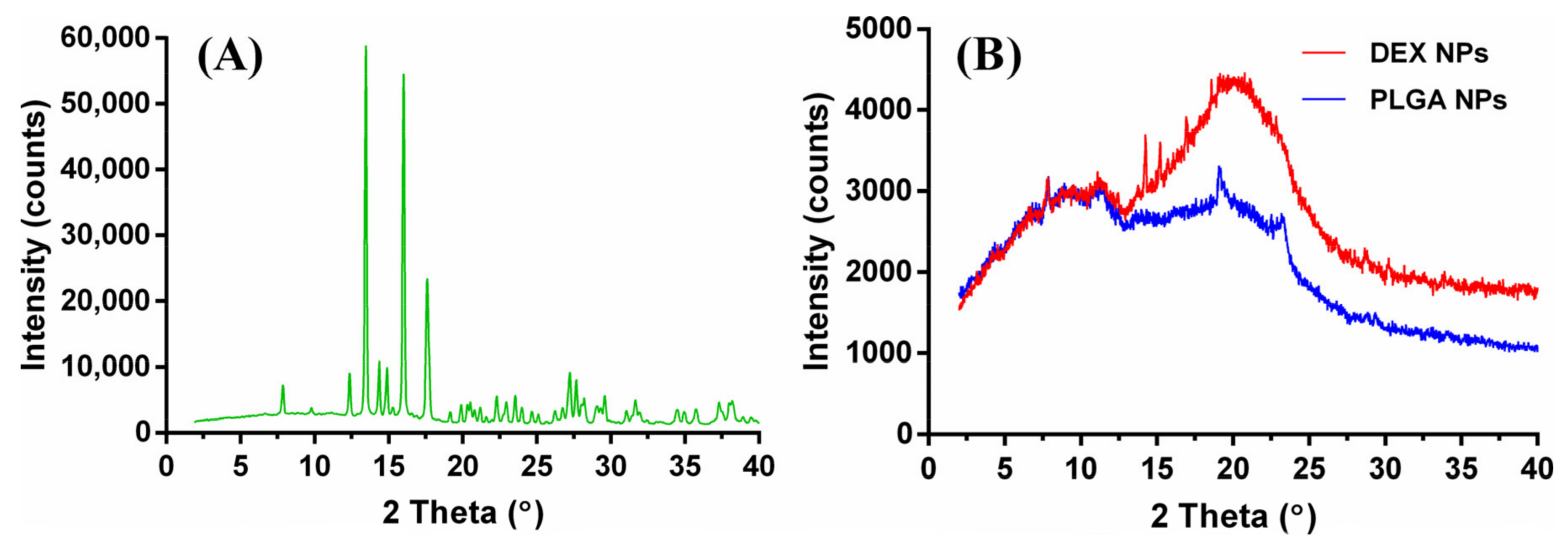
XRD patterns of DEX (**A**), DEX NPs, and blank PLGA NPs (**B**).

XRD analysis was used to evaluate the physical state of DEX and the degree of its incorporation in the polymeric NPs. Figure 4 shows XRD patterns of DEX, DEX NPs, and blank PLGA NPs. The presence of numerous distinct peaks in the DEX diffractogram implies its crystalline nature, as reported in the literature [34,35]. The diffraction pattern of DEX NPs shows a broad hump in the region of 10–25°. This is indicative of the amorphous polymeric matrix, and the presence of some characteristic peaks of DEX indicates the presence of it in the NPs. The intensity of these peaks was reduced, suggesting that a significant loss of crystallinity of DEX occurred while being incorporated in the polymeric matrix. It is dispersed at a molecular state within the nanoparticles [36,37]. These findings are in agreement with the DSC thermogram of DEX NPs, in which the DEX crystalline peak at 255 °C was not observed. This indicates a degree of amorphization of the drug within the PLGA matrix.

### 3.2. Fabrication of SA-MNs and NP-MNs

**Figure 5:**
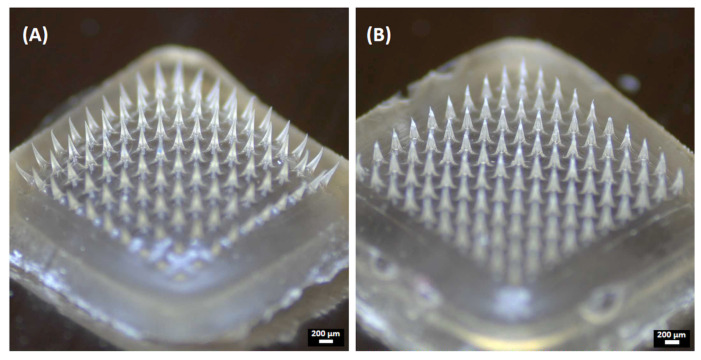
Representative images of the obtained microneedles: (**A**) SA-MNs and (**B**) NP-MNs.

Sodium alginate (SA) is one of the most widely used materials in the biomedical field [38,39]. SA at a concentration of 4% (*w*/*v*) was chosen from preliminary tests for the fabrication of dissolving MNs. In the case of NP-MNs, the obtained nanoparticles were encapsulated into the tips of SA MNs, being concentrated at the tips rather than being dispersed in the base layer to provide more efficient delivery by decreasing drug wastage [40,41]. The MNs were fabricated as a 10 × 10 array (Figure 5) through a vacuum-deposition micromolding method, as depicted in Figure 1.

**Figure 6:**
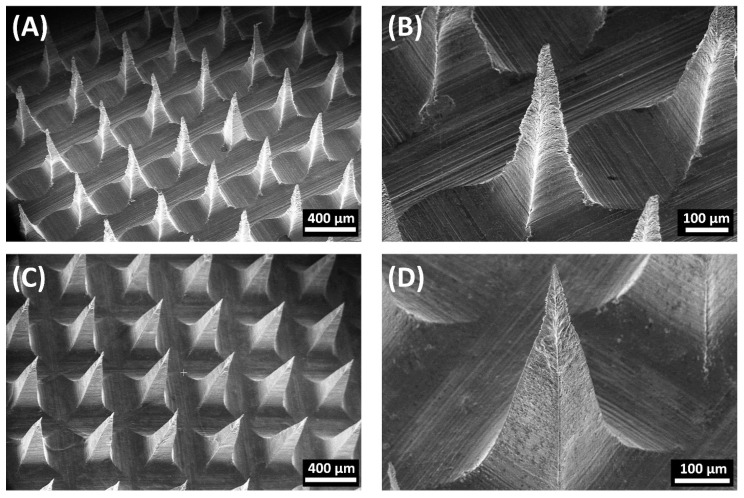
Representative SEM images of the obtained microneedles: (**A**) SA-MNs, (**B**) Close-up view of SA-MNs, (**C**) NP-MNs, and (**D**) Close-up view of NP-MNs.

When characterized by SEM, the MN formulations had a quadrangular pyramidal shape and were uniformly distributed on the substrate (Figure 6). SA-MNs and NP-MNs were successfully formed with dimensions of approximately 500 μm height and 200 μm width of the base, as represented in Table 2. Moreover, the SEM images confirmed sharper tips and a smoother surface of the NP-MNs compared to SA-MNs.

### 3.3. Insertion Capabilities of MN Array in Parafilm

**Figure 7:**
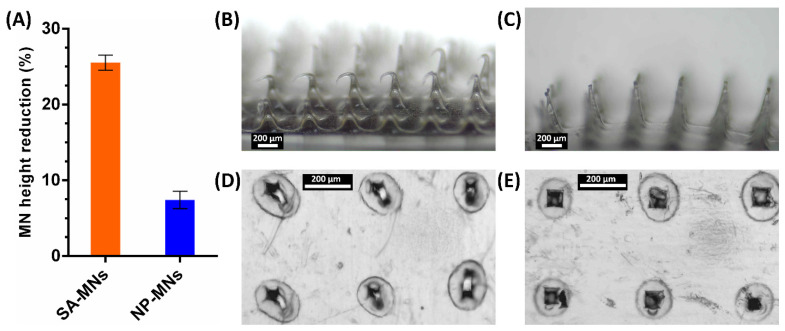
Insertion test in the parafilm skin simulant model. MN height reduction for MNs after insertion test using the parafilm skin simulant model (**A**), results expressed as means ± s.d., *n* = 10. SA-MNs (**B**) and NP-MNs (**C**) acquired with the optical microscope after the insertion test. Representative microscopic images of the first layer of parafilm into which SA-MNs (**D**) and NP-MNs (**E**) were inserted.

To evaluate the insertion properties of MNs and to analyze MN insertion depth, commercial parafilm was used as a skin simulant for preliminary assessment instead of biological tissues [42]. In this test, 8 layers of parafilm (thickness of 140 ± 10 µm each) were stacked together for MN insertion by manual pressure (using a thumb) to imitate the practical use in clinical settings. After the insertion of MNs on a stack of parafilm layers, the layers were separated and visualized under an optical microscope, and the MN height reduction was evaluated. Both SA-MNs and NP-MNs created 100 pores only in the first parafilm layer, indicating that the MN insertion depth is approximately 140 µm. This suggests that MNs readily pierce the outermost layer of the skin, the stratum corneum (~50 μm thickness), and allow MN insertion into the epidermis [42]. Figure 7 shows images of the first layer of parafilm into which SA-MNs and NP-MNs were inserted, in which NP-MNs created square-shaped pores, while SA-MNs created pores with a less defined shape, presumably due to the different surface roughness and the structural change that occurred during the insertion process. Then the MNs were visualized after the insertion test, showing the bending of SA-MNs and an insignificant change in the morphology of NP-MNs, in which the mean percentage reduction in the height of SA-MNs and NP-MNs was 25.5%, and 7.4%, respectively. This demonstrates that NP-MNs became slightly compressed and were mechanically stronger than SA-MNs. MNs loaded with blank PLGA NPs were used as controls and tested in the same model. Interestingly, they created 100 square-shaped holes only in the first parafilm layer (Figure S4) and exhibited a mean percentage of height reduction of 12.9 ± 1.8%. This is in line with our hypothesis that the incorporation of PLGA NPs into the SA-MNs improves their mechanical strength and penetration performance. A plausible explanation for the difference in percentage of height reduction between DEX NP-MNs and MNs loaded with blank PLGA NPs could be the increased density of nanoparticles, due to the incorporation of DEX and the lower diameter of DEX NPs compared to blank PLGA NPs (Table 1).

### 3.4. Mechanical Strength of MNs

**Figure 8:**
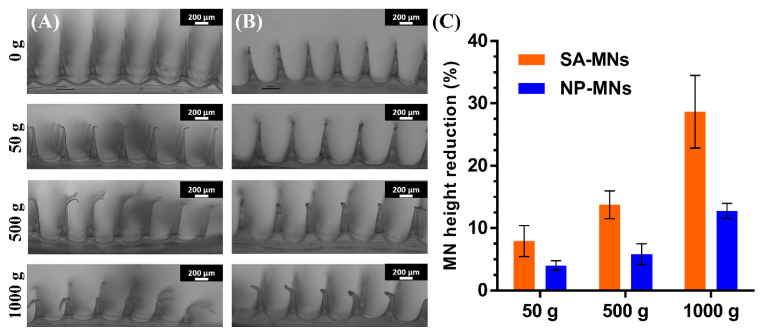
The mechanical strength of SA-MNs and NP-MNs after applying different weights: Optical images of the deformation of (**A**) SA-MNs and (**B**) NP-MNs under weights of 50, 500, and 1000 g, scale bar 200 μm. (**C**) MN height reduction for MNs after compression: results expressed as means ± s.d., *n* = 10.

To further shed light on the mechanical strength of the MNs, the morphological changes of MN tips against static force were microscopically observed by placing different weights on the top of the MN arrays for 5 min, and the height reduction percentages were calculated. As shown in Figure 8, the sharp tips of MNs exhibited reduction and deformation in the vertical direction as a function of the static force ranging from 50 g (~4.9 mN/needle) to 1000 g (~98 mN/needle). The MNs showed adequate mechanical strength, and no fractures or broken MNs were noted, even though they were pressed by a 1000-g weight [43]. Significant morphological changes and differences in the average percent height reduction of SA-MNs and NP-MNs were observed after applying different weights (Figure 8C), ascertaining that the incorporation of DEX NPs into the MNs improved their mechanical strength, in agreement with the results of the parafilm insertion test.

### 3.5. Dissolution Test of MNs In Ex Vivo Chicken Skin and Agarose Gel

**Figure 9:**
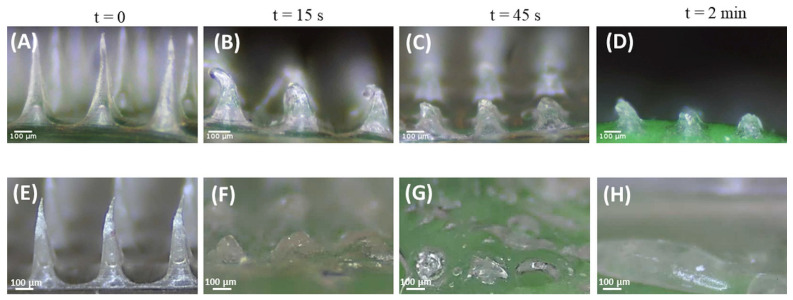
Ex-vivo and in vitro dissolution studies of NP-MNs: Representative images of NP-MNs before (**A**,**E**) and after dissolution in ex vivo chicken for 15 (**B**), 45 (**C**), and 120 s (**D**) and agarose gel after 15 (**F**), 45 (**G**), and 120 s (**H**). Scale bar 100 µm.

The dissolving capability of the polymeric tips affects the release rate of NPs from MNs. Herein, ex vivo chicken skin, frequently used for the characterization of microneedle arrays [22,44,45], and agarose gel, mimicking the mechanical properties of human skin [39,42,46], were employed to determine the time for morphological changes to occur in the structure of MNs after insertion. The NP-MNs were gradually dissolved within 2 min after insertion into ex vivo chicken skin, with a bending already visible after 15 s. The needle height of NP-MNs decreased to ca. 50%, 40%, and 30% in 15, 45, and 120 s, respectively (Figure 9B–D). This indicates that the NP-loaded segments of MNs were dissolved within 15 s. In contrast, after insertion into the agarose gel, the tips of NP-MNs were significantly dissolved after 15 s, and the needle height declined to 25% (Figure 9F). These findings confirm the fast dissolving feature of NP-MNs and indicate that the dissolution of NP-MNs is not expected to be the rate-limiting step in the transdermal delivery of DEX-NPs.

Due to the high-water solubility of SA, the MNs could dissolve very quickly and thoroughly. The presence of a surfactant plays a vital role in formulating dissolving MNs [47]. It is presumed that solutol HS 15 affected the wetting of the polymeric matrix, thus increasing the water penetration through the MNs and eventually decreasing the overall dissolution time. These results suggest that the recommended application time of the MN patch can be set as >15 s [48]. Additionally, once the MNs dissolve in the skin and the drug-loaded NPs are released, the base can be readily rinsed with water, promoting skin recovery and restoring its barrier function. This is another important aspect for preventing potential skin infections due to the presence of microchannels created by the MNs [2,4].

Despite the significant value of these skin-simulant models for the characterization of MNs, there are some limitations to be considered, such as the lack of pharmacopeial standards for MN-based products [49], the absence of validation of in vitro methods to characterize the dissolution and release profiles of therapeutics from MNs containing nanosystems [24], and the differences between the human skin and skin simulants used in this study, like their composition and water content [42,50,51,52].

### 3.6. In Vitro Drug Release Studies

**Figure 10:**
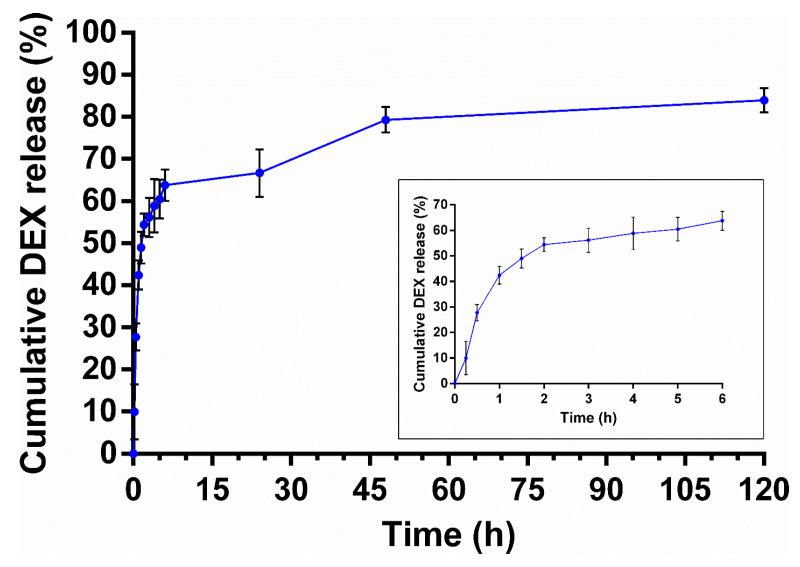
In vitro DEX release from PLGA NPs (pH 7.4, 37 °C). Inset shows the drug release profile over the initial 24 h. Values are mean ± s.d. of four experiments.

Based on the dissolution study findings, in vitro release studies of DEX from PLGA NPs were performed using a dialysis bag in phosphate buffer saline (pH 7.4) at 37 °C for 120 h. The release data presented in Figure 10 indicate that the release of DEX was biphasic, and a burst drug release was observed in the first 6 h followed by sustained release over 120 h. The results are comparable to previous studies in which DEX was incorporated into the PLGA matrix. The initial burst release could be a result of DEX molecules that are weakly bound/adsorbed to the NP surface [26,53,54]. This release profile would effectively suppress both acute and chronic inflammation [55].

The main challenges of the current topical treatment modalities for inflammatory skin diseases, such as psoriasis and atopic dermatitis, are the lack of selectivity in delivering the drug to the inflammation site, side effects associated with steroids, inadequate skin penetration, and patient adherence [56,57]. Nanoparticle-based drug delivery systems are beneficial in overwhelming the limitations of conventional dosage forms by enhancing the local drug effect. Nanomedicines allow for achieving controlled drug release and skin targeting by improving retention and drug localization in the skin layers, as well as protection of the drug against chemical or physical changes and, consequently, improve patient compliance by reducing application frequency [56,58,59].

Recent studies have highlighted the beneficial use of polymeric nanoparticles for anti-inflammatory topical treatment due to their ability to form a drug reservoir, retaining the drug locally at the site of action [60,61]. It was also shown that polymeric nanocarriers have a higher potential than lipid vehicles for local therapy of inflammatory skin diseases with minimum systemic absorption due to their rigid and stable structure [57,62]. Try et al. report that small PLGA NPs (below 100 nm) can accumulate selectively in the viable epidermis and hair follicles in two atopic dermatitis animal models, while larger PLGA NPs (about 300 nm) remain at the epidermis surface [63]. The findings are in line with previous findings reported by Abdel-Mottaleb et al. in which polymeric NPs made of ethyl cellulose were tested [64].

This suggests that the delivery system proposed in this study, based on the combination of minimally invasive dissolving MNs with PLGA NPs, can be foreseen as an interesting strategy to treat various inflammatory skin diseases by means of increased patient compliance, controlled drug release, improved safety, and efficacy of topical steroid therapy.

## 4. Conclusions

The work presented demonstrates a useful step-by-step strategy, based on a modified vacuum-deposition micromolding method to fabricate rapidly dissolving MNs loaded with biodegradable NPs for the treatment of skin diseases. DEX-loaded PLGA NPs were successfully prepared, with acceptable drug loading content exhibiting a controlled release profile. The incorporation of DEX-NPs into the tips of dissolving MNs, made of SA, significantly improves their mechanical strength and insertion capabilities, as demonstrated by the skin-simulant parafilm model and static force tests. The MN dissolution study following application to ex vivo chicken skin and skin mimicking agarose gel reveals that the NP-loaded segments of MNs have been dissolved within 15 s. Taken together, the combination of dissolving MNs and biodegradable NPs offers an interesting minimally invasive approach to bypass the limitations of conventional topical treatment of skin diseases, allowing for self-administration, increased patient compliance, and controlled drug release. Thus, the potential of this delivery system warrants further ex vivo and in vivo investigations.

## Figures and Tables

**Table 1:** Physicochemical properties, encapsulation efficiency, and drug loading content of blank and DEX-loaded PLGA NPs (*n* = 3, mean ± s.d.).

Formulation	Mean Diameter (nm)	Polydispersity Index (PDI)	Zeta Potential (mV)	Encapsulation Efficiency (%)	Drug Loading Content (%)
DEX NPs	93.7 ± 5.10	0.27 ± 0.04	−27.5 ± 3.31	80 ± 0.6	9.1 ± 0.1
Blank PLGA NPs	116 ± 1.92	0.13 ± 0.03	−35.6 ± 1.63	-	-

**Table 2:** Summary of microneedle dimensions (*n* = 10, mean ± s.d.).

Formulation	Base (μm)	Height (μm)
SA-MN	199.9 ± 1.3	500.2 ± 0.4
NP-MNs	200.3 ± 1.9	500.2 ± 0.1

## Data Availability

Not applicable.

## Supplementary Materials

The following supporting information can be downloaded at: https://www.mdpi.com/article/10.3390/pharmaceutics15020526/s1, Figure S1: Calibration curve of DEX in distilled water containing 5% DMSO at 242 nm. Figure S2: Calibration curve of DEX in PBS (pH = 7.4) at 242 nm. Figure S3: FTIR spectrum of Solutol HS 15. Figure S4: Images of MNs loaded with blank PLGA NPs (A) before and (C) after the insertion test. (B) Representative microscopic image of holes created on the first parafilm layer.
