# Supplementary material for: Rapidly Dissolving Microneedles for the Delivery of Steroid-Loaded Nanoparticles Intended for the Treatment of Inflammatory Skin Diseases
Source: Pharmaceutics. 2023 Feb 4;15(2):526. doi: 10.3390/pharmaceutics15020526 (PMC9967926; doi:10.3390/pharmaceutics15020526)
Supplement: Supplementary file 1 [file pharmaceutics-15-00526-s001.zip › pharmaceutics-2097778-supplementary.pdf]

Supplementary Materials

Rapidly Dissolving Microneedles for the Delivery of Steroid-Loaded Nanoparticles Intended for the Treatment of Inflammatory Skin Diseases

Hala Dawud and Aiman Abu Ammar *

Department of Pharmaceutical Engineering, Azrieli College of Engineering Jerusalem, 26 Yaakov Shreibom Street, Ramat Beit HaKerem, Jerusalem 9103501, Israel

* Correspondence: aimanab@jce.ac.il; Tel.: +972-2-6591835

Contents:

Figure S1. Calibration curve of DEX in distilled water containing 5% DMSO at 242 nm.

Figure S2. Calibration curve of DEX in PBS (pH=7.4) at 242 nm.

Figure S3. FTIR spectrum of Solutol HS 15.

Figure S4. Images of MNs loaded with blank PLGA NPs (A) before and (C) after the insertion test. (B) Representative microscopic image of holes created on the first parafilm layer.

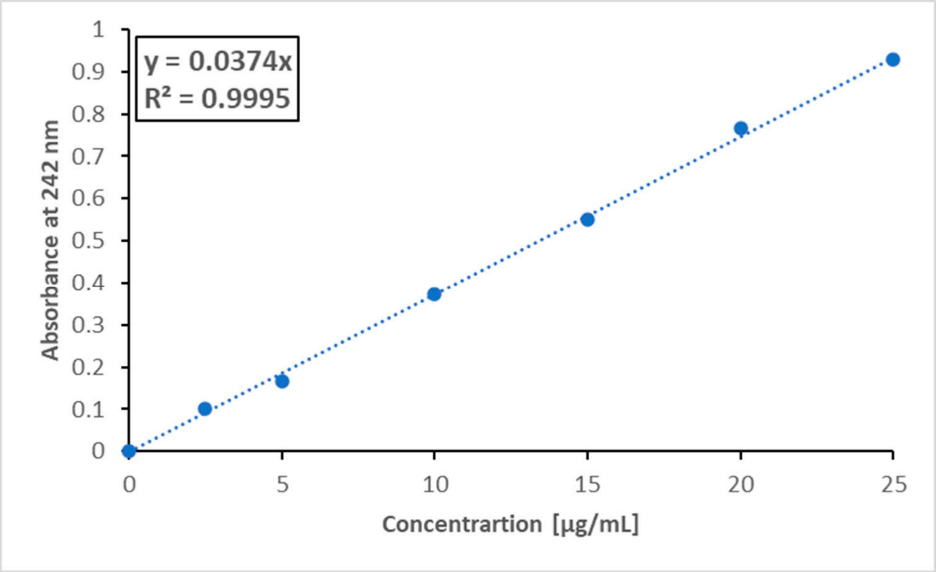

Figure S1. Calibration curve of DEX in distilled water containing 5% DMSO at 242 nm.

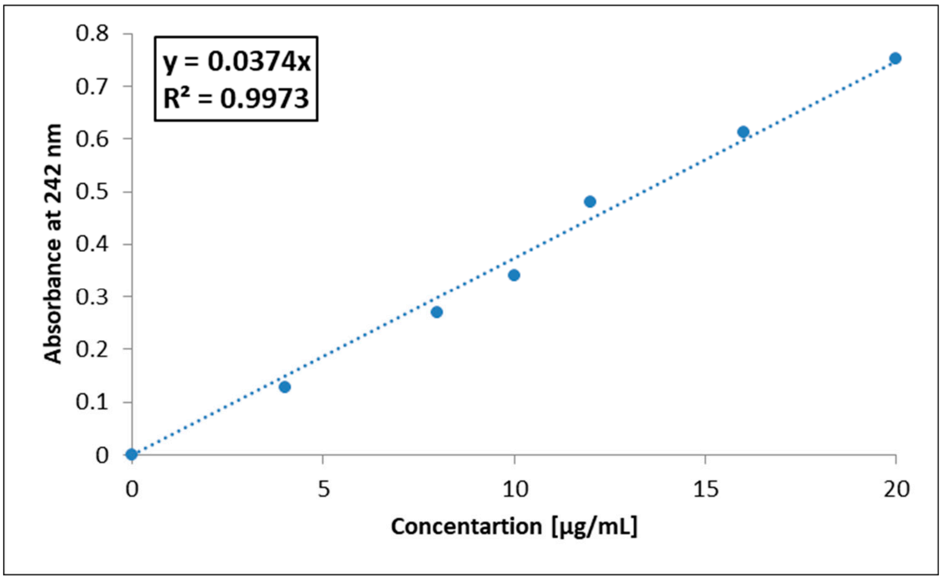

Figure S2. Calibration curve of DEX in PBS (pH=7.4) at 242 nm.

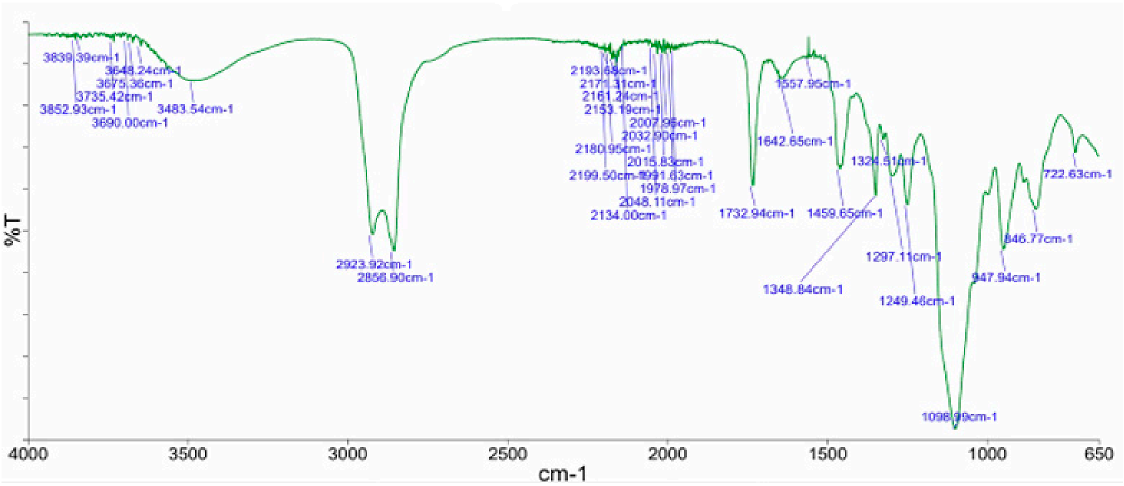

Figure S3. FTIR spectrum of solutol HS 15.

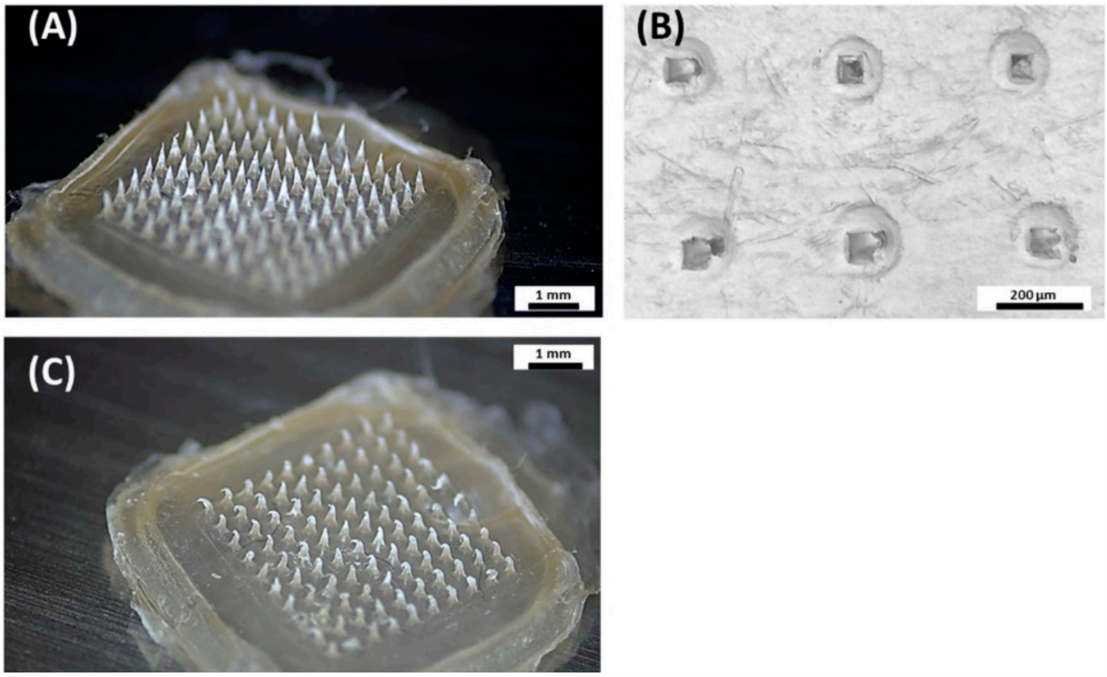

Figure S4. Images of MNs loaded with blank PLGA NPs (A) before and (C) after the insertion test. (B) Representative microscopic image of holes created on the first parafilm layer.
